# Comparison of two nutritional protocols in body re-composition of resistance-trained participants

**DOI:** 10.1007/s00421-026-06209-6

**Published:** 2026-04-06

**Authors:** Salvador Vargas-Molina, Alejandro García-Palumbo, Manuel García-Sillero, Diego A. Bonilla, Jorge L. Petro, Alan A. Aragon, Brad J. Schoenfeld, Javier Benítez-Porres

**Affiliations:** 1 Department of Human Physiology, Physical Education and Sport, Faculty of Medicine, University of Malaga, Málaga, Spain; 2 Research Group in Sports Nutrition (DBSS-Nut), Dynamical Business & Science Society—DBSS International SAS, Bogotá, Colombia; 3 Universidad Maimonides, Buenos Aires, Argentina; 4 Research Group in Physical Activity, Sports and Health Sciences (GICAFS), Universidad de Córdoba, Monteria, Colombia; 5 Fit Advancement, LLC, Chatsworth, California USA; 6 Department of Exercise Science and Recreation, CUNY Lehman College, Bronx, NY USA; 7 Institute of Biomedical Research of Málaga (IBIMA BIONAND Platform), Málaga, Spain; 8 CIBER Physiopathology of Obesity and Nutrition (CIBEROBN), Carlos III Health Institute, Madrid, Spain

**Keywords:** Body composition, Hypertrophy, Fat free mass, Lean body mass, Isocaloric diet, Energy deficit

## Abstract

**Purpose:**

This study aimed to compare the effects of two high-protein nutritional protocols (isocaloric and moderate energy deficit), combined with a structured resistance training program, to a control condition that followed the same training program without dietary intervention or supervision, on body recomposition outcomes. One protocol generated a caloric deficit and the other protocol used an isocaloric diet.

**Methods:**

Thirty participants (23.0 ± 3.4 years, 174.3 ± 8.0 cm, 80.3 ± 16.0 kg, 26.3 ± 4.5 kg·m^− 2^) were randomized into one of three nutritional conditions: an isocaloric diet group (ISO = 10), energy deficit group (DEF = 10) or a control group without nutritional supervision. Participants in ISO and DEF performed resistance training 4 days a week for 10 weeks and consumed nutritional protocols that contained the same amounts of protein but with different amounts of total calories. Body composition was assessed by dual X-ray densitometry at baseline and post-study.

**Results:**

DEF reduced fat mass (FM; Δ =  − 2.94 kg; p < 0.001) and fat-free adipose tissue (FFAT; Δ =  − 0.47 kg; p = 0.016), while ISO showed smaller decreases (FM: Δ =  − 1.41 kg; p = 0.051; FFAT: Δ =  − 0.25 kg; p = 0.054). Fat-free mass (FFM) increased in both ISO (Δ = 0.97 kg; p < 0.001) and DEF (Δ = 1.04 kg; p = 0.009), as did FFM adjusted for FFAT (FFM − FFAT; ISO Δ = 1.22 kg; p = 0.002; DEF Δ = 1.50 kg; p < 0.001), whereas the control group showed no meaningful changes (all p ≥ 0.778). The Time × Group interaction was significant for FFM (p = 0.034) and FFM − FFAT (p = 0.006), but not for FM or FFAT (p > 0.05).

**Conclusions:**

Both a moderate energy deficit and a maintenance-calorie high-protein diet can elicit body recomposition when compared to habitual practice, suggesting that elevated protein intake (2.5 g·kg⁻^1^·d⁻^1^) may facilitate simultaneous improvements in fat mass and FFM. These findings challenge the traditional model of energy balance.

**Supplementary Information:**

The online version contains supplementary material available at 10.1007/s00421-026-06209-6.

## Introduction

Studies investigating changes in body composition have primarily focused on the increase in muscle mass (Vargas et al. 2019) or the reduction of fat mass (FM) (Vargas-Molina et al. 2023), with program variables and nutritional strategies directed towards one objective or the other. To this end, the laws of thermodynamics have played a crucial role, dictating that optimal muscle tissue growth requires an energy surplus (Iraki et al. 2019; Helms et al. 2014) while adipose tissue reduction necessitates an energy deficit (Iraki et al. 2019; Helms et al. 2014).

However, in many cases, people aspire to increase muscle mass while simultaneously reducing adipose tissue, a phenomenon known as body recomposition (BR) (Barakat et al. 2020). It has been speculated that the simultaneous process of increasing skeletal muscle cross-sectional area and reducing adipose tissue occurs only in obese or sedentary individuals (Coker et al. 2012; Ormsbee et al. 2018) or older adults (Hooshmand-Moghadam et al. 2023; Mastalerz et al. 2024). However, some studies have also reported BR in resistance-trained participants (Haun et al. 2018; Kreipke et al. 2015).

The optimal range of protein intake for optimizing muscle hypertrophy has been estimated to be between 1.6 g∙kg^− 1^ d^− 1^ to 2.2 g∙kg^− 1^ d^− 1^, (Iraki et al. 2019). However, if the goal is to reduce adipose tissue, an increase in protein intake of 1.8 g∙kg^− 1^ d^− 1^ to 2.7 g∙kg^− 1^ d^− 1^ of body weight (Phillips and Van Loon 2011) or 2.3 g∙kg^− 1^ d^− 1^ to 3.1 g∙kg^− 1^ d^− 1^ of fat-free mass (FFM) (Helms et al. 2014) has been proposed due to protein’s satiating effect (Belza et al. 2013) and its increased thermic effect (Ravn et al. 2013). However, the primary benefit of increasing protein intake in the diet appears to be its role in preservation of muscle mass, as indicated in a recent systematic review and meta-regression that determined an intake of 1.9–3.2 g∙kg^− 1^ d^− 1^ BM/d or 2.5–4.2 g∙kg^− 1^ d^− 1^ FFM/d was most effective at preserving lean mass in non-obese, resistance trained subjects sustaining hypocaloric conditions (Refalo et al. 2025). It is important to note that when the goal is to reduce adipose tissue, it is common to increase the total training volume (more sets per muscle group for resistance training and a greater duration of cardio) or training density (supersets or high intensity interval training), in addition to decreasing energy consumption. In this regard, our lab previously investigated two groups of trained women with an average energy restriction of 29.5 kcal/kg-FFM/d, performing concurrent training with 170 min of cardiovascular exercise per week and employing supersets. Fat mass and FFM were assessed by dual-energy X-ray densitometry. Both groups consumed an average protein dose of 2.6 g∙kg^− 1^ d^− 1^ of BM. Results showed no loss of FFM in either protocol while both groups reduced fat mass (Vargas-Molina et al. 2023).

Protein requirements tend to increase during periods of energy restriction, and higher protein intakes have been associated with favorable outcomes that support BR (Longland et al. 2016; Campbell et al. 2018). Although exceeding the minimum protein threshold is not necessary for muscle mass gains (Schoenfeld and Aragon 2018), a high protein intake may promote muscle mass maintenance. Body recomposition has been explored in studies comparing protocols with differing levels of protein intake. Therefore, we aimed to examine the potential differences in BR when matching protein intake above generally established thresholds with participants following either an isocaloric diet or sustaining a modest energy deficit. We hypothesized that both high-protein protocols would induce greater body recomposition compared to the control condition, with potential differences between the isocaloric and energy deficit approaches..

## Methods

### Trial design

Participants were randomized (www.randomizer.org) into three groups: (1) a slight energy deficit diet group (DEF = 10, 2 women and 8 males); (2) a normocaloric diet group (ISO = 10, 1 woman and 9 males); and (3) a control group (CG = 10, 1 woman and 9 males) that maintained their habitual diet. All groups performed the same four-day-per-week resistance training program for 10 weeks. However, only the DEF and ISO groups received supervision and nutritional guidance throughout the intervention, whereas the control group completed the training program independently without supervision.

### Participants

We recruited 30 participants between 18 and 35 years of age with more than one consecutive year of resistance training experience (4 women and 26 men; resistance training experience: 1.5 ± 0.5 years; age: 23.0 ± 3.4 years; height: 174.3 ± 8.0 cm; weight: 80.3 ± 16.0 kg; body mass index: 26.3 ± 4.5 kg·m^− 2^). The participants were informed of the possible risks of the experiment and signed an informed consent form. The research protocol was approved by the Ethics Committee of the XXX (blind) in accordance with the ethical guidelines of the Declaration of Helsinki (WMO 2013). Participants were eligible for inclusion if they: (i) were between 18 and 35 years of age; (ii) had at least one consecutive year of resistance training experience; (iii) were training a minimum of three times per week prior to enrollment; and (iv) were free from musculoskeletal injuries or medical conditions that could interfere with resistance training or body composition assessment.

Participants were excluded if they: (i) were following a structured nutritional intervention at the time of recruitment; (ii) self-reported the use of anabolic steroids or other performance-enhancing substances within the previous year; (iii) were taking medications known to affect metabolism or body composition; (iv) had experienced body mass fluctuations greater than 3 kg in the three months prior to the study; or (v) failed to comply with the prescribed intervention or missed more than 10% of the training sessions (for ISO and DEF groups).

Sample size determination was guided by pragmatic considerations. Specifically, given the substantial logistical demands and resource constraints inherent to conducting supervised nutritional and resistance training interventions, we sought to recruit the maximum number of participants feasible (Lakens 2022) to optimize statistical power.

### Intervention procedures

#### Exercise protocol

**Fig. 1:**
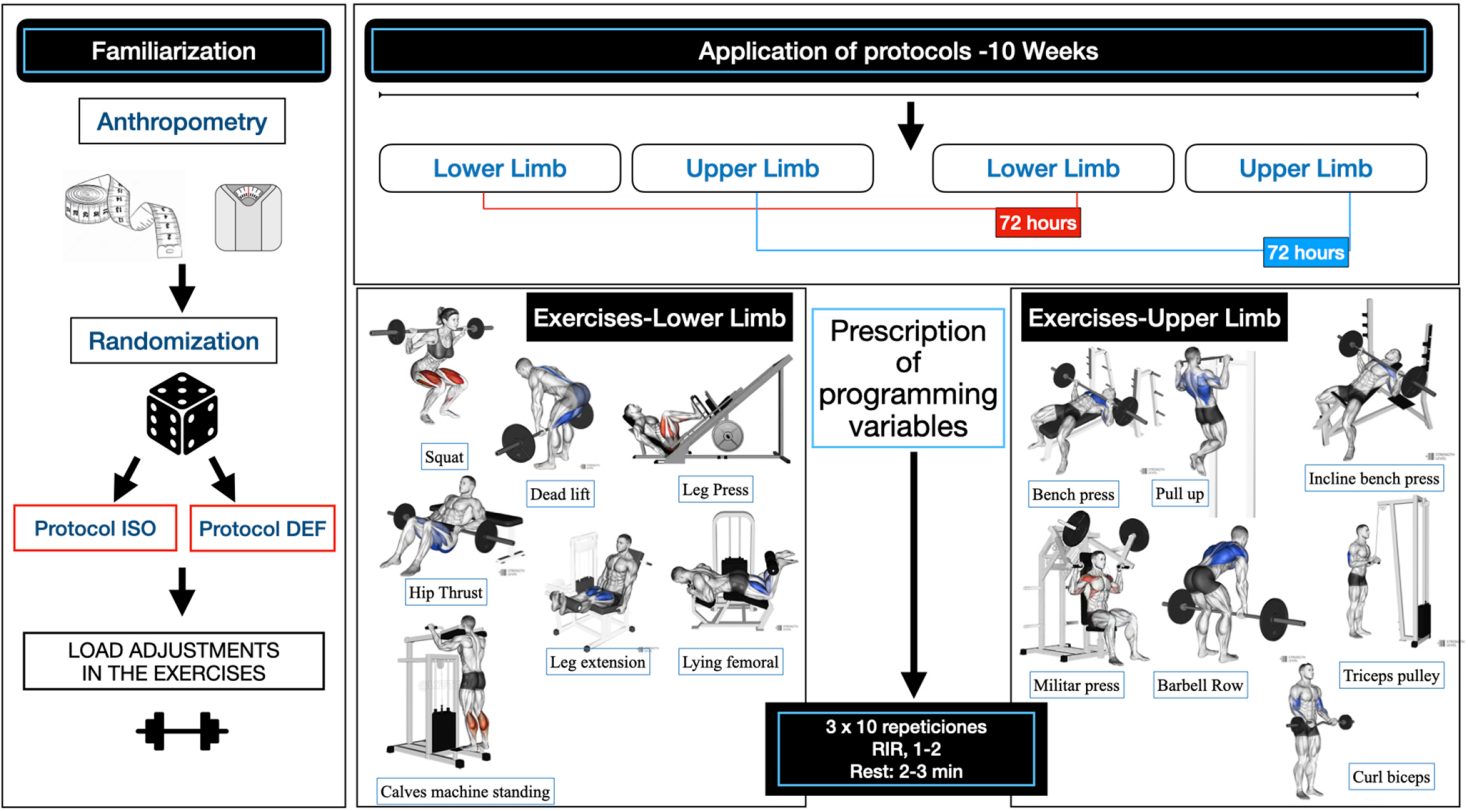
Training protocol

Following a one-week familiarization period, participants underwent a ten-week training program, with all sessions supervised by two researchers to ensure protocol adherence and exercise fidelity. Training was carried out four days per week, consisting of two sessions targeting the torso muscles and two sessions for the legs, with 72 h of recovery between upper and lower limb sessions (see Fig. 1). Participants performed three sets of 10 repetitions for each exercise, with loads adjusted to maintain 1 or 2 repetitions in reserve (RIR, 1–2) and a recovery interval of between two and three minutes between sets and exercises. The starting load was the the same as that used during the familiarization week, and the loads were adjusted accordingly throughout the study. In this case, if a participant failed to complete 10 repetitions in a set, the load was reduced in subsequent sets. Conversely, if participants exceeded 10 repetitions, the load was increased. Repetitions were performed with a controlled speed of execution lasting approximately one second for both the concentric and eccentric actions.

#### Nutritional protocols

Participants assigned to the ISO group were prescribed maintenance caloric intake, while those in the DEF group were instructed to maintain an energy intake 250 kcal below estimated maintenance requirements. Maintenance calories were calculated using the equation of Cunningham (Cunningham 1980) and values were adjusted to individual physical activity levels. Both DEF and ISO protocols were instructed to consume 2.5 g∙kg^− 1^ d^− 1^ of protein, with 40% of total calories from carbohydrates and the balance of calories from fats (we prescribed consumption of primarily monounsaturated and polyunsaturated fats). The rationale for implementing a modest deficit is based on the tendency for aggressive deficits (promoting > 0.5–1.0% decrease in bodyweight per week) to significantly decrease lean mass, especially in lean, trained subjects (Ruiz-Castellano et al. 2021). Thus, a modest deficit would theoretically increase the likelihood of BR instead of merely promoting fat loss. Prescribed daily energy and macronutrient targets for the ISO and DEF groups are summarized in Table 1.

**Table 1:** Prescribed energy and macronutrient targets for the ISO and DEF dietary protocol

Group	Protein	CHO	Lipids	Caloric Intake	
	(g·kg⁻^1^·day⁻^1^)	(g·kg⁻^1^·day⁻^1^)	(g·kg⁻^1^·day⁻^1^)	(g·kg⁻^1^·day⁻^1^)	(kcal·day⁻^1^)
ISO	2.49 ± 0.74	3.16 ± 0.72	1.04 ± 0.23	31.99 ± 7.50	2565.5 ± 277.17
DEF	2.50 ± 0.72	2.54 ± 0.74	0.89 ± 0.36	28.17 ± 8.19	2262.3 ± 459.91

Values are presented as mean ± SD of the prescribed targets. Macronutrients are expressed relative to body mass (g·kg⁻^1^·day⁻^1^); energy is expressed as kcal·kg⁻^1^·day⁻^1^ and kcal·day⁻^1^. These dietary prescriptions applied only to the ISO and DEF groups; the Control group received no dietary prescription or nutritional supervision. ISO, isocaloric; DEF, moderate energy deficit; CHO, carbohydrates; BM, body mass

Participants were instructed to consume five meals per day, aligned with their habitual daily routines. Ongoing nutritional guidance and communication were provided by a doctor specializing in sports nutrition. Each participant received an individualized dietary plan that provided flexible food options to promote greater adherence. Dietary monitoring and adjustments were managed using specialized software (Nutrium, Braga, Portugal), which facilitated comprehensive tracking and compliance throughout the intervention.

### Measurements

#### Body composition

Total and regional body composition was estimated using dual-energy x-ray absorptiometry (DXA). All participants were instructed to arrive for testing in a fasted state (no food or caloric beverages for at least 8 h, with water allowed) and to avoid physical activity during the 12 h prior to the assessment. They were also asked to empty their bladder immediately before the body composition measurement. In addition, for female participants, DXA scans were scheduled 7 days after the onset of menstruation, both pre- and post-intervention, to minimize potential fluctuations in body mass from fluid retention associated with hormonal variations (Rosenfeld et al. 2008).

A certified technician conducted all DXA assessments. Computer algorithms (APEX 5.6.0.7 software version, Hologic Horizon A, Waltham, MA) were employed to distinguish bone and soft tissue, detect borders, and analyze regional demarcations. All participants wore athletic clothing and removed materials that could attenuate the x-ray beam, including jewelry and underwear containing wire. Densitometer calibration was verified daily with a standard calibration block provided by the manufacturer. Coefficient of variation values ​​were less than 2% for all whole-body and segmental body composition measurements, including bone mineral density (g/cm^2^), mineral content (g), FM (%), FM (g), lean mass (g), and total body mass (g). The DXA unit was calibrated with phantoms according to the manufacturer’s guidelines each day before assessment.

Based on the assumption that adipose tissue is composed of approximately 85% fat, fat-free adipose tissue (FFAT) was estimated using the following equation: (FM / 0.85) × 0.15 (Heymsfield et al. 2002). Subsequently, FFM excluding FFAT (FFM-FFAT) was calculated and reported following the methodology previously described by our group (Bonilla et al. 2021).

#### Volume load

Training volume was calculated as the product of sets, repetitions, and load (sets × reps × load). Given that the number of sets and repetitions was matched between groups, differences in training volume were attributed solely to variations in the loads lifted. Supervising researchers recorded the loads lifted in each set for later analysis based on research from our lab (Vargas-Molina et al. 2024).

#### Repetition maximum (1-RM)

Repetition maximum (RM) was assessed in the squat (SQ) and bench press (BP) performed on Technogym equipment (Cesena, Italy). Participants refrained from strength exercise for 48 h prior to assessment. Testing followed the usual protocol by our working group (Romance et al. 2019) that began with a 7–10 min warm-up followed by a specific warm-up set of 12–15 repetitions at 40% of perceived 1-RM. Participants then performed 2–3 approach sets between 60–80 of the 1-RM. As many 1-RM attempts at maximum weight were performed as necessary until reaching maximum weight. SQ and BP technique was monitored by two of the investigators. Subjects were required to reach parallel in the 1-RM SQ; confirmation of squat depth was obtained by a research assistant positioned laterally to the subject to ensure accuracy. Successful 1-RM Bench Press was achieved if the subject displayed a five-point body contact position (head, upper back, and buttocks firmly on the bench with both feet flat on the floor) and executed full-elbow extension. 1-RM SQ testing was conducted before 1-RM BP with a 7-min rest period separating tests. Participants then performed as many attempts as necessary until repetition failure,

### Statistical analysis

Continuous data are summarized as mean ± standard deviation when approximately normal, and as median (interquartile range) otherwise. Normality was assessed with the Shapiro–Wilk test. Baseline between-group differences in age, stature, body mass, and BMI were examined with robust one-way ANOVA based on 20% trimmed means, and effect sizes were expressed as the robust explanatory ES with bootstrap 95% confidence intervals (CIs). Within-group pre–post change was analyzed from an estimation perspective (Ho et al., 2019), prioritizing effect magnitude and precision; we calculated paired Hedges’ g (g) against baseline with 95% CIs) obtained via bias-corrected and accelerated (BCa) bootstrap (5000 resamples). As a complementary, legacy reference, two-sided permutation t-tests were also reported (5000 label shuffles); effect sizes (ES) were interpreted using conventional absolute thresholds (trivial < 0.20, small 0.20–0.49, medium 0.50–0.79, and large ≥ 0.80) (Cohen, 1988). Mixed two-way repeated-measures ANOVA (Time × Group) was applied to body composition outcomes (FM, FFM, FFAT, FFM − FFAT), strength outcomes (1-RM Bench Press and Squat), and weekly training volume loads (LBLT and UBTL). In these models, we report p-values and partial eta-squared (η^2^p). As a descriptive guide, η^2^p values of 0.01, 0.06, and 0.14 have been described as small, medium, and large, respectively; these cutoffs are field-dependent and should be interpreted alongside effect estimates and their uncertainty (Gaskill and Garner 2020; Lakens 2013). Pairwise post hoc comparisons of estimated marginal means were adjusted with Bonferroni. An α = 0.05 was set for all analyses. Analyses were performed in IBM SPSS Statistics v27.0 (IBM Corp., Armonk, NY), and in Python v3.12.11 (Python Software Foundation), using dabest-python v2025.03.27.

## Results

**Fig. 2:**
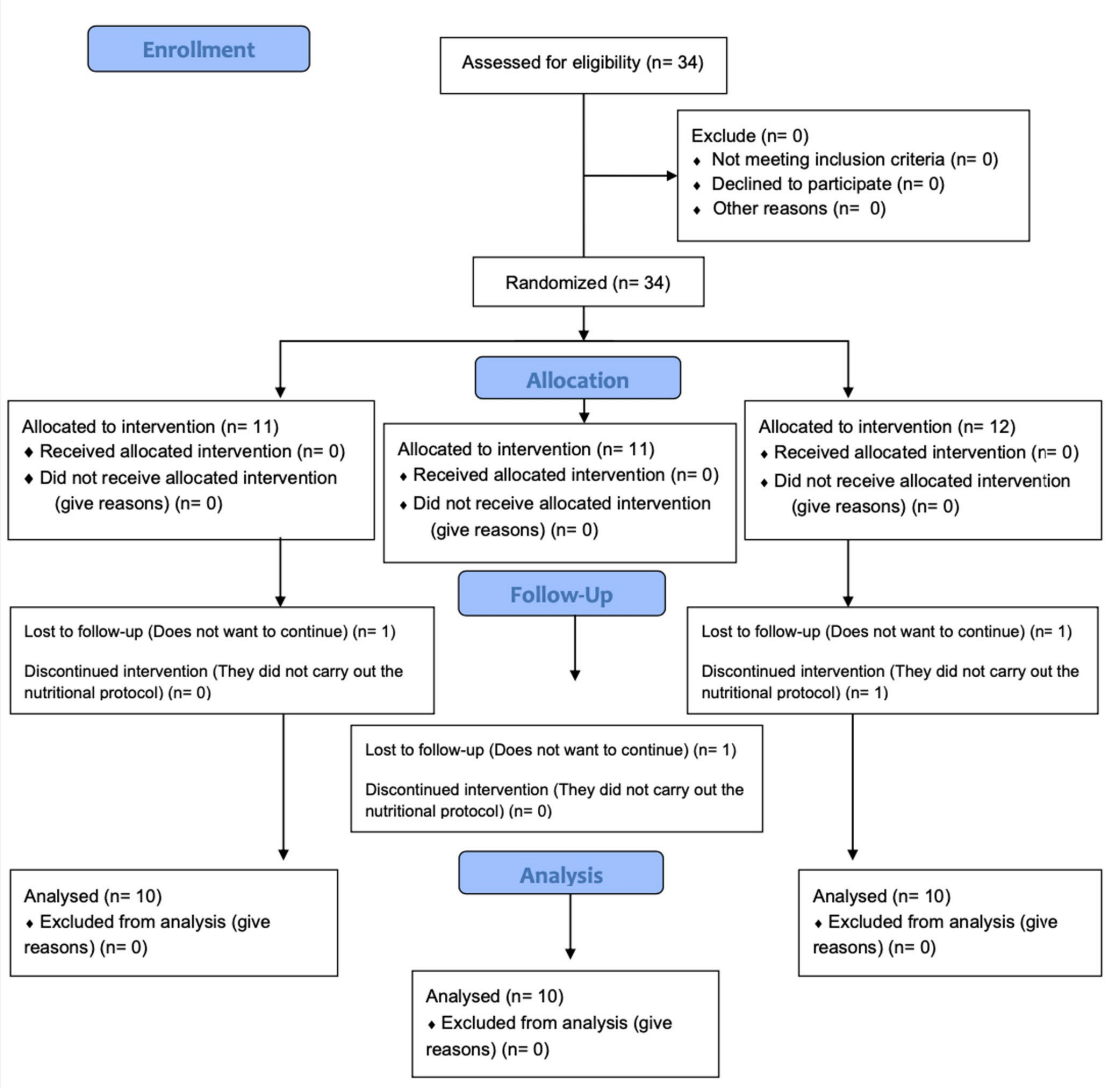
Consolidated standards of reporting trials (CONSORT) flow diagram

Participant flow is summarized in the CONSORT diagram (Fig. 2). Thirty-four participants were randomized across the study’s three arms and 30 subjects ultimately completed the intervention, with an equal number of participants per group (n = 10 each).

Table 2 shows the baseline characteristics of the three groups (CON, ISO, DEF); there were no between-group statistical differences in age, stature, body mass, or BMI (all *p* ≥ 0.109), and effect sizes were trivial (ES ≤ 0.09).

**Table 2:** Characteristics of participants by group

	CON (n = 10)	ISO (n = 10)	DEF (n = 10)	p	ES
Age (y)	24.2 ± 3.7	23.6 ± 4.0	21.2 ± 1.1	0.117	0.52
Stature (cm)	174.5 ± 7.8	174.6 ± 8.5	173.9 ± 8.5	0.601	0.21
Body mass (kg)	80.5 ± 15.3	80.2 ± 17.6	80.3 ± 16.7	0.676	0.21

Values are presented as mean ± SD.* p* values correspond to between-group comparisons performed with with robust one-way ANOVA; ES, effect size: CG, control; ISO, isocaloric; DEF, caloric deficit; BMI, body mass index

**Fig. 3:**
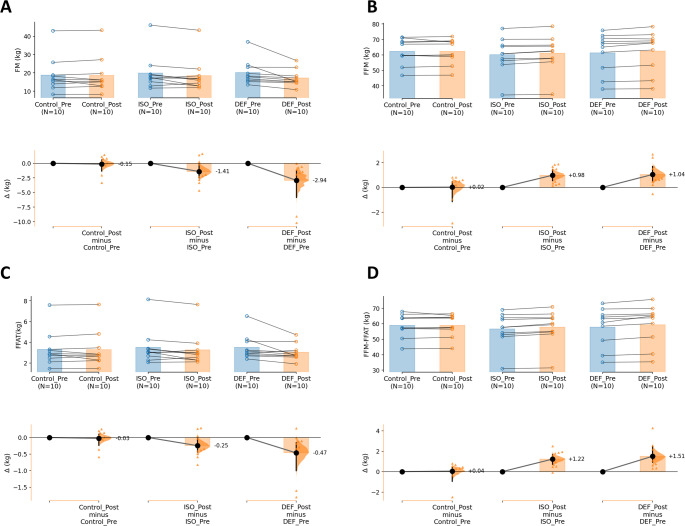
Cumming estimation plots of body composition. **A** Fat mass (FM); **B** Fat-free mass (FFM); **C** Fat-free adipose tissue (FFAT); **D** Fat-free mass minus FFAT (FFM − FFAT). In each panel, the raw data is plotted on the upper axes, and each paired set of observations is connected by a line; on the lower axes, each paired mean difference is plotted as a bootstrap sampling distribution. Mean differences are depicted as dots; 95% confidence intervals are indicated by the ends of the vertical error bars

In body composition, the DEF group reduced FM (Δ =  − 2.94 kg; g =  − 0.49 (− 0.89 – − 0.26); p < 0.001) and FFAT (Δ =  − 0.47 kg; g =  − 0.43 (− 0.88 – − 0.15); p = 0.016), while FFM (Δ = 1.04 kg; g = 0.08 (0.02–0.17); p = 0.009) and FFM–FFAT (Δ = 1.50 kg; g = 0.11 (0.05–0.25); p < 0.001) increased; in the ISO group, FM and FFAT showed small decreases (FM: Δ =  − 1.41 kg; g =  − 0.14 (− 0.46–0.17); p = 0.051; FFAT: Δ =  − 0.25 kg; g =  − 0.14 (− 0.49–0.18); p = 0.054), whereas FFM and FFM–FFAT increased (FFM: Δ = 0.97 kg; g = 0.08 (0.02–0.17); p < 0.001; FFM–FFAT: Δ = 1.22 kg; g = 0.11 (0.03–0.25); p = 0.002); in the Control group, FM and FFAT showed no change (FM: Δ =  − 0.15 kg; g =  − 0.01 (− 0.35–0.08); p = 0.782; FFAT: Δ =  − 0.03 kg; g =  − 0.01 (− 0.35–0.08); p = 0.778), while FFM (Δ = 0.02 kg; g = 0.00 (− 0.19–0.06); p = 0.975) and FFM–FFAT (Δ = 0.04 kg; g = 0.01 (− 0.20–0.06); p = 0.942) remained stable (Table 3; Fig. 3). Regarding strength, 1-RM Bench Press increased in Control (Δ = 4.38 kg; g = 0.12 (0.03–0.40); p < 0.001) and DEF (Δ = 4.10 kg; g = 0.12 (0.03–0.40); p < 0.001), whereas ISO showed no change (Δ = 1.00 kg; g = 0.04 (− 0.04–0.25); p = 0.245); for 1-RM Squat, variations were small: Control showed a slight increase (Δ = 3.88 kg; g = 0.22 (0.04–0.46); p < 0.001), and ISO and DEF showed trivial effects (ISO: Δ = 1.30 kg; g = 0.08 (− 0.05–0.28); p = 0.135; DEF: Δ = 2.33 kg; g = 0.09 (0.00–0.27); p < 0.001) (Table 3). Individual pre-to-post responses for each participant are shown in Fig. 3 A–D.

**Table 3:** Pre–post changes in body composition and strength across the study groups

	Group	Time	Time x Group	Group	*g* (95% CI)		Main effects and interaction^*^		
							Time	Time x Group	Group
FM (kg)	Control	18.8 ± 9.6	18.7 ± 10.0	-0.15 (-1.30 to 0.55)	− 0.01 (− 0.35 to 0.08)	0.782	0.007 (0.27)	0.277 (0.10)	0.984 (0.00)
	ISO	20.0 ± 9.8	18.5 ± 9.2	-1.41 (-2.62 to -0.15)	− 0.14 (-0.46 to 0.17)	0.051			
	DEF	20.2 ± 6.8	17.2 ± 4.6	-2.94 (-5.73 to -1.25)	− 0.49 (− 0.89 to − 0.26)	< 0.001			
FFM (kg)	Control	62.3 ± 8.4	62.4 ± 8.1	0.02 (-1.03 to 0.48)	0.00 (− 0.19 to 0.06)	0.975	0.006 (0.28)	0.034 (0.25)	0.923 (0.01)
	ISO	60.2 ± 11.5	61.1 ± 11.5	0.97 (0.54 to 1.41)	0.08 (0.02–0.17)	< 0.001			
	DEF	61.4 ± 13.0	62.4 ± 13.2	1.04 (0.45–1.64)	0.08 (0.02–0.17)	0.009			
FFAT (kg)	Control	3.3 ± 1.7	3.3 ± 1.8	-0.03 (-0.23 to 0.10)	− 0.01 (− 0.35 to 0.08)	0.778	0.016 (0.22)	0.439 (0.07)	0.976 (0.00)
	ISO	3.5 ± 1.7	3.3 ± 1.6	-0.25 (-0.46 to – 0.02)	− 0.14 (− 0.49 to 0.18)	0.054			
	DEF	3.5 ± 1.2	3.0 ± 0.8	-0.47 (-0.98 to –0.14)	− 0.43 (− 0.88 to − 0.15)	0.016			
FFM − FFAT (kg)	Control	59.0 ± 7.5	59.1 ± 7.2	0.04 (-0.91 to 0.41)	0.01 (− 0.20 to 0.06)	0.942	< 0.001 (0.46)	0.006 (0.35)	0.914 (0.01)
	ISO	56.6 ± 10.7	57.9 ± 10.8	1.22 (0.75–1.70)	0.11 (0.03–0.25)	0.002			
	DEF	57.9 ± 12.7	59.4 ± 13.0	1.50 (0.93–2.39)	0.11 (0.05–0.25)	< 0.001			
1-RM Bench Press (kg)	Control	88.1 ± 22.2	92.5 ± 23.7	4.38 (2.12–6.12)	0.12 (0.03–0.40)	< 0.001	0.002 (0.34)	0.235 (0.11)	0.597 (0.04)
	ISO	76.8 ± 21.1	77.8 ± 22.8	1.00 (-0.80 to 3.60)	0.04 (− 0.04 to 0.25)	0.245			
	DEF	85.5 ± 32.7	89.6 ± 35.0	4.10 (1.40–9.90)	0.12 (0.03–0.40)	< 0.001			
1-RM Squat (kg)	Control	117.1 ± 17.2	121.0 ± 16.5	3.88 (1.38–6.38)	0.22 (0.04–0.46)	< 0.001	0.002 (0.35)	0.341 (0.09)	0.348 (0.08)
	ISO	105.5 ± 16.4	106.8 ± 14.9	1.30 (-0.50 to 3.30)	0.08 (− 0.05 to 0.28)	0.135			
	DEF	107.2 ± 23.5	109.6 ± 25.1	2.33 (0.56–5.22)	0.09 (0.00–0.27)	< 0.001			

Values are mean ± SD; Δ denotes the change (post–pre); Δ and paired Hedges’ g are reported with 95% BCa bootstrap CIs (5,000 resamples); permutation p values were obtained from 5,000 label reshuffles. * p-value (partial η^2^p) for *Time*, *Time* × *Group*, and *Group.* DEF, slight energy deficit; FFAT, fat-free adipose tissue; FFM, fat free mass; FFM–FFAT, FFM adjusted for FFAT; FM, fat mass; ISO, isocaloric

To place these within-group changes in a between-group context, we applied a mixed repeated-measures ANOVA (Table 3). There was a main effect of time across all variables (all p ≤ 0.016; η^2^p = 0.22–0.46); for FM and FFAT, neither a Time × Group interaction (FM: p = 0.277; η^2^p = 0.10; FFAT: p = 0.439; η^2^p = 0.07) nor a main effect of group (FM: p = 0.984; η^2^p = 0.00; FFAT: p = 0.976; η^2^p = 0.00) was detected; by contrast, the interaction was statistically significant for FFM (p = 0.034; η^2^p = 0.25) and FFM–FFAT (p = 0.006; η^2^p = 0.35), consistent with the increases observed in ISO and DEF relative to Control. No Time × Group interactions were found for 1-RM Bench Press (p = 0.235; η^2^p = 0.11) or 1-RM Squat (p = 0.341; η^2^p = 0.09), although a main effect of time was present for both outcomes (both p = 0.002; η^2^p = 0.34–0.35). For body composition and strength, Bonferroni-adjusted pairwise comparisons are shown in Supplementary Table S1; none reached significance after adjustment.

For total weekly training volume load, both lower-body (LBLT) and upper-body (UBTL) loads rose from pre to post across groups (see Table 4). In LBLT, Control increased (Δ = 2.39 (1.82–2.99); g = 1.75 (1.13–2.90); p < 0.002), ISO showed a larger gain (Δ = 28.00 (19.80–35.00); g = 2.81 (1.51–4.87); p < 0.001), and DEF improved (Δ = 2.24 (1.54–3.00); g = 1.16 (0.72–1.72); p < 0.001). A mixed repeated-measures ANOVA (Time × Group) indicated statistically significant main effects of Time, Group, and their interaction (all p < 0.001; η^2^p = 0.74, 0.77, 0.98, respectively). The same pattern was observed for UBTL, with larger magnitudes: Control (Δ = 8.5 ± 1.4; g = 7.27 (5.43–9.15); p < 0.001), ISO (Δ = 12.5 ± 2.5; g = 6.69 (5.33–9.23); p < 0.001), and DEF (Δ = 5.9 ± 1.5; g = 3.15 (1.71–5.09); p < 0.001). Consistently, the mixed ANOVA also showed statistically significant Time, Time × Group, and Group effects in UBTL (all p < 0.001; η^2^p = 0.963, 0.69, 0.99). Post-hoc tests again favored ISO over DEF and Control: ISO–Control 28.40 (95% CI 26.89–29.92; p < 0.001), ISO–DEF 25.51 (95% CI 24.00–27.03; p < 0.001), and DEF–Control 2.89 (95% CI 1.41–4.36; p < 0.001); full pairwise results are reported in Supplementary Table S2.

**Table 4:** Pre–post changes in lower- and upper-body training-load volume across the study groups

	Group	Pre	Post	Δ (95% CI)	*g* (95% CI)	*p*	Main effects and interaction^*^		
							Time	Time x Group	Group
LBLT (t)	Control	116.2 ± 1.4	118.5 ± 1.2	2.39 (1.82–2.99)	1.75 (1.13–2.9)	< 0.002	< 0.001 (0.74)	< 0.001 (0.77)	< 0.001 (0.98)
	ISO	166.7 ± 2.6	194.7 ± 13.2	28.00 (19.80–35.00)	2.81 (1.51–4.87)	< 0.001			
	DEF	136.4 ± 1.5	138.7 ± 2.1	2.24 (1.54–3.00)	1.16 (0.718–1.72)	< 0.001			
UBTL (t)	Control	68.1 ± 0.6	76.5 ± 1.4	8.47 (7.66–9.27)	7.27 (5.43–9.15)	< 0.001	< 0.001 (0.963)	< 0.001 (0.69)	< 0.001 (0.99)
	ISO	94.5 ± 1.5	107.1 ± .0	12.50 (10.90–14.00)	6.69 (5.33, 9.23)	< 0.001			
	DEF	72.2 ± 1.6	78.1 ± 2.0	5.91 (4.98–6.75)	3.15 (1.71–5.09)	< 0.001			

Values are mean ± SD; Δ denotes the change (post–pre); Δ and paired Hedges’ g are reported with 95% BCa bootstrap CIs (5,000 resamples); permutation p values were obtained from 5,000 label reshuffles. * p-value (partial η^2^p) for *Time*, *Time* × *Group*, and *Group.* DEF, slight energy deficit; ISO, isocaloric; LBLT, lower-body training load; UBTL, upper-body training load

## Discussion

Our study aimed to investigate the effect of two nutritional protocols with an equated protein intake (2.5 g∙kg^− 1^ d^− 1^) but different total energy consumption on measures of body composition. While participants in ISO consumed their estimated maintenance calories, the DEF protocol restricted daily energy intake by 250 kcal. Results indicated that both ISO and DEF experienced a loss of FM (− 1.41 (− 2.62 – − 0.15) kg vs − 2.94 (− 5.73 – − 1.25) kg, respectively), but only the reduction in the DEF group reached statistical significance and it was of greater magnitude. Regarding FFM, both groups gained relatively similar amounts FFM (0.97 (0.54–1.41) kg and 1.04 (0.45–1.64) kg for ISO and DEF, respectively). When applying the FFM correction factor, both protocols elicited statistically significant increases, with the ISO group gaining 1.22 (0.75–1.70) kg and the DEF group gaining 1.50 (0.93–2.39) kg.

Traditional nutritional dogma often promotes an oversimplified (and often misinterpreted) CICO (calories in/calories out) model that fails to consider the different energy densities of fat mass versus lean mass. The energy content of lean mass is 7.6 MJ/kg (1815.2 kcal/kg), while fat contains 39.5 MJ/kg (9434.4 kcal/kg) (Hall 2008). Changes in these tissues can occur in bidirectionally and independently of the sustained state of energy balance, as observed repeatedly in research demonstrating the BR phenomenon (Barakat et al. 2020). Specifically, lean mass can be increased in hypocaloric conditions and fat mass can be decreased in hypercaloric conditions. For example, Haun et al. (2018) demonstrated a decrease in FM in resistance individuals performing a high-volume resistance training program combined with high protein intake (> 2.0 g/kg/d) despite participants reportedly consuming an energy surplus of 500 cal/d. Moreover, Longland et al. (2016) demonstrated increases in FFM with concomitant reductions in FM in a cohort of untrained young men consuming a hypocaloric diet, with higher protein intakes (2.4 g/kg/d) showing more favorable body composition changes compared to lower intakes (1.2 g/kg/d). In line with these findings, our results demonstrated significant reductions in fat mass in the DEF group and increases in FFM in both ISO and DEF conditions, despite differing caloric intakes. This supports the notion that body recomposition may occur independently of a strict energy balance model when protein intake is sufficiently elevated and resistance training volume is progressively increased. A potential mechanism underlying these adaptations may involve enhanced muscle protein synthesis coupled with improved nutrient partitioning, favoring lean mass accretion while facilitating fat mass reduction.

Several studies have evaluated resistance training combined with differing protein intakes and total caloric intake. Campbell et al., (2018) compared consumption of 2.5 g_**·**_kg^− 1^_**·**_d^− 1^ vs. 0.9 g_**·**_kg^− 1^_**·**_d^− 1^ of protein combined with RT in a cohort of trained women. FFM gain and fat reduction favored the group that consumed the higher protein diet despite self-reportedly consuming > 400 more calories/d than the lower protein group (Campbell et al. 2018). These results were partly expected, as the lower threshold for protein intake to optimize increases in muscle mass has been estimated to be between 1.6 g_**·**_kg^− 1^_**·**_d^− 1^ and 2.2 g_**·**_kg^− 1^_**·**_d^− 1^ (Iraki et al. 2019), and in this case, the lower protein group consumed well below these ranges. Similarly, in the present study, both intervention groups consumed 2.5 g·kg⁻^1^·d⁻^1^ of protein, above the suggested threshold for optimizing hypertrophy, which may explain the increases in FFM observed even in the absence of a caloric surplus. Unlike Campbell et al., total caloric intake was intentionally controlled between ISO and DEF, allowing us to better isolate the role of energy availability while maintaining high protein intake.

Other research has investigated protein intakes substantially higher than the recommended range for optimal muscular gains. For example, Antonio et al., showed no statistical differences in FFM in a cohort of resistance trained men and women when consuming 4.4 g·kg⁻^1^·d⁻^1^ vs. 1.8 g·kg⁻^1^·d⁻^1^ of protein (Antonio et al. 2014). This same research group conducted two subsequent studies. In the first, they evaluated 2.3 g·kg⁻^1^·d⁻^1^ vs. 3.4 g·kg⁻^1^·d⁻^1^ of protein, with no statistical differences found between groups (Antonio et al. 2015). Another study from the same lab compared 2.6 g·kg⁻^1^·d⁻^1^ vs. 3.3 g·kg⁻^1^·d⁻^1^ of protein; again, no statistically significant differences were observed in body composition between groups (Antonio et al. 2016). Nevertheless, in the context of an energy deficit, it is necessary to increase protein intake beyond the Recommended Dietary Allowance (0.8 g·kg⁻^1^·d⁻^1^), as this strategy may support gains in FFM (Chapman et al. 2021) or help preserve it under conditions of severe or progressive energy restriction, provided it is accompanied by resistance training (Vargas-Molina et al. 2023). Our findings partially align with these observations, as no extreme protein intakes were necessary to induce favorable changes in body composition. This may suggest that once protein intake surpasses an adequate threshold (e.g., ≥ 2.2–2.5 g·kg⁻^1^·d⁻^1^), further increases may not provide additional benefits, particularly when resistance training volume is sufficient to stimulate adaptation. Mechanistically, this could reflect a plateau effect in muscle protein synthesis once maximal stimulation is achieved.

In all four of the aforementioned studies, the group that consumed more protein also reportedly consumed more total calories. Moreover, total calories were not controlled; participants either ate ad libitum or were instructed to maintain their usual intake of carbohydrates and fats (Antonio et al. 2014, 2015, 2016; Campbell et al. 2018). For example, participants in the high protein group in the study by Antonio et al. (Antonio, 2014) consumed ~ 800 kcal/d more than the lower protein group, yet no statistically significant between-group differences were observed for total mass, FM, or FFM. This finding led the authors to conclude that a high-calorie, high-protein diet does not increase FM. In fact, it is speculated that body recomposition occurs with precise nutritional adjustment, with protein intake in ranges between 2.4 g·kg⁻^1^·d⁻^1^ and 3.4 g·kg⁻^1^·d⁻^1^ (Vecchio 2021). The present study extends this literature by controlling both protein intake and caloric intake across conditions, while implementing a standardized resistance training program. Notably, training volume load increased significantly in all groups, but the magnitude of improvement was greatest in the ISO group, which may have contributed to the observed body recomposition effects. The interaction between elevated protein intake, progressive overload, and energy availability likely played a central role in driving these adaptations.

From a performance standpoint, all conditions showed increases in 1-RM values; however, the results were only statistically significant in the Control and DEF groups. Interestingly, the control group had the greatest effect size for strength outcomes despite training without supervision. It should be noted that 1-RM testing can be influenced by numerous factors including fatigue, sleep quality, stress, motivation, etc. and assessments can vary by as much as 18% on a daily basis (Pareja-Blanco et al. 2017). Therefore, given the lack of statistical significance in the ISO group without a logical explanation for between-group differences, the observed results may be spurious.

### Limitations

Our study has several limitations of note. First, although we took care to ensure that our DXA device was properly calibrated on a daily basis, the use of multi-compartment models such as the 4C would yield greater accuracy for body composition assessment (Graybeal et al. 2020). Moreover, despite DXA’s reputation as a criterion/gold standard method for assessment of FFM, direct measures such as magnetic resonance imaging, computed tomography and ultrasonography could provide better insights into actual changes in skeletal muscle tissue (Delmonico et al. 2008; Snijders et al. 2015).

Second, the capacity for experiencing BR inevitably diminishes alongside an individual’s training status and proximity to genetic potential for muscular size and leanness. Novice trainees (or highly deconditioned individuals) with excess body fat are primed for substantial degrees of BR, at least at the outset of their programs. In contrast, advanced trainees with low body fat have a diminished capacity for BR and may be better served by focusing on one goal at a time (thus strategically implementing either a caloric surplus or deficit). For intermediate-level trainees who comprise a broad bridge between these two ends of the spectrum, the potential for BR needs to be investigated on an individual basis. In our study, which involved a predominantly male sample (4 women, 26 men), the subjects in the ISO and DEF groups averaged 24.8 & 24.7% body fat at baseline, respectively. This level of body fat is sufficiently high to facilitate BR. In the case of a sample with greater training experience and muscular development and/or lower body fat levels, the BR results may have been less robust.

Third, it should be noted that increases in protein intake can still result in body fat gain (alongside lean mass gain) under hypercaloric conditions. This has been demonstrated in tightly controlled metabolic ward conditions where all food & beverages were provided to the subjects, including a protein intake of approximately 3.0 g/kg in the high-protein group (Bray et al. 2012). One important caveat is that the referenced findings (Bray et al. 2012) were derived from sedentary, untrained individuals, markedly differing from the free-living conditions and resistance training protocols used in our study to achieve BR.

Fourth, actual dietary intake during the intervention was not quantified; thus, adherence to the prescribed macronutrient and energy targets cannot be confirmed quantitatively and findings should be interpreted as effects of the prescribed protocols under supervised guidance.

Finally, our work is limited by the common logistical challenges inherent in this area of study, including a relatively small sample size and short trial duration. Future research should investigate longer-term effects of similar experimental treatments and perhaps include hypercaloric conditions to provide additional insights into the topic. Moreover, caution should be applied when generalizing our results to adult populations aside from our sample of healthy, young, predominantly male subjects.

## Conclusions

Our investigation demonstrates that a moderate caloric deficit (-250 kcal) combined with a protein intake of 2.5 g·kg⁻^1^·d⁻^1^ combined with regular resistance training promotes a loss of body fat while simultaneously increasing FFM. Both experimental groups reduced their body fat, but DEF showed greater losses compared to ISO (-2.94 kg vs. -1.41 kg, respectively) and a slightly greater gain in lean mass (+ 1.04 kg vs. + 0.97 kg after FFM correction, respectively). Despite the modest absolute difference, however, the ES of the lean mass gains were nearly identical between the ISO and DEF groups. These results challenge the traditional CICO model and suggest that BR is enhanced by consuming a modest caloric deficit while maintaining a high protein intake. It remains to be determined the extent to which recomposition can occur at varying levels of energy and protein intake.

## Electronic Supplementary Material

Below is the link to the electronic supplementary material.

Supplementary Material 1

## Data Availability

The data presented in this study are available on request from the corresponding authors.

## Abbreviations

ISO: Isocaloric
DEF: Deficit
FM: Fat mass
FFM: Fat free mass
BR: Body recomposition
BM: Body mass
CG: Control group
RIR: Repetition in reserve
DXA: Dual-energy x-ray absorptiometry
FFAT: Fat-free adipose tissue
RM: Repetition maximum
SQ: Squat
BP: Bench press
CIs: Confidence intervals
BCa: Bias-corrected and accelerated
EF: Effect sizes
BMI: Body mass index
LBLT: Both lower-body
UBTL: Upper-body
g: Grams
kg: Kilograms
kcal: Kilocalories
CICO: Calories in calories out
