# Supplementary material for: Comparison of two nutritional protocols in body re-composition of resistance-trained participants
Source: Eur J Appl Physiol. 2026 Apr 6;126(7):4019–30. doi: 10.1007/s00421-026-06209-6 (PMC13380550; doi:10.1007/s00421-026-06209-6)
Supplement: Supplementary file 1 — Supplementary Material 1 [file 421_2026_6209_MOESM1_ESM.docx]

**Tabla S1.** Bonferroni-adjusted pairwise comparisons of body composition and strength

| Measure | | | Mean Difference (I-J) | Std. Error | p^a^ | 95% Confidence Interval for Difference^a^ | |  |  |
| --- | --- | --- | --- | --- | --- | --- | --- | --- | --- |
|  |  |  |  |  |  | Lower Bound | Upper Bound |  |  |
| **FM (kg)** | Control | ISO | -0.183 | 4.067 | 1.000 | -10.65 | 10.28 |  |  |
|  |  | DEF | 0.500 | 4.166 | 1.000 | -10.22 | 11.22 |  |  |
|  | ISO | Control | 0.183 | 4.067 | 1.000 | -10.28 | 10.65 |  |  |
|  |  | DEF | 0.683 | 3.940 | 1.000 | -9.46 | 10.82 |  |  |
|  | DEF | Control | -0.500 | 4.166 | 1.000 | -11.22 | 10.22 |  |  |
|  |  | ISO | -0.683 | 3.940 | 1.000 | -10.82 | 9.46 |  |  |
| **LST (kg)** | Control | ISO | 2.179 | 5.465 | 1.000 | -11.89 | 16.25 |  |  |
|  |  | DEF | 1.417 | 5.599 | 1.000 | -12.99 | 15.83 |  |  |
|  | ISO | Control | -2.179 | 5.465 | 1.000 | -16.25 | 11.89 |  |  |
|  |  | DEF | -0.762 | 5.294 | 1.000 | -14.39 | 12.86 |  |  |
|  | DEF | Control | -1.417 | 5.599 | 1.000 | -15.83 | 12.99 |  |  |
|  |  | ISO | 0.762 | 5.294 | 1.000 | -12.86 | 14.39 |  |  |
| **FFAT (kg)** | Control | ISO | -0.031 | 0.718 | 1.000 | -1.88 | 1.82 |  |  |
|  |  | DEF | 0.118 | 0.735 | 1.000 | -1.78 | 2.01 |  |  |
|  | ISO | Control | 0.031 | 0.718 | 1.000 | -1.82 | 1.88 |  |  |
|  |  | DEF | 0.149 | 0.695 | 1.000 | -1.64 | 1.94 |  |  |
|  | DEF | Control | -0.118 | 0.735 | 1.000 | -2.01 | 1.78 |  |  |
|  |  | ISO | -0.149 | 0.695 | 1.000 | -1.94 | 1.64 |  |  |
| **FFM-FFAT (kg)** | Control | ISO | 2.211 | 5.202 | 1.000 | -11.18 | 15.60 |  |  |
|  |  | DEF | 1.300 | 5.329 | 1.000 | -12.41 | 15.01 |  |  |
|  | ISO | Control | -2.211 | 5.202 | 1.000 | -15.60 | 11.18 |  |  |
|  |  | DEF | -0.911 | 5.039 | 1.000 | -13.88 | 12.06 |  |  |
|  | DEF | Control | -1.300 | 5.329 | 1.000 | -15.01 | 12.41 |  |  |
|  |  | ISO | 0.911 | 5.039 | 1.000 | -12.06 | 13.88 |  |  |
| **BP (kg)** | Control | ISO | 13.013 | 12.845 | 0.963 | -20.05 | 46.07 |  |  |
|  |  | DEF | 5.368 | 13.158 | 1.000 | -28.50 | 39.23 |  |  |
|  | ISO | Control | -13.013 | 12.845 | 0.963 | -46.07 | 20.05 |  |  |
|  |  | DEF | -7.644 | 12.442 | 1.000 | -39.67 | 24.38 |  |  |
|  | DEF | Control | -5.368 | 13.158 | 1.000 | -39.23 | 28.50 |  |  |
|  |  | ISO | 7.644 | 12.442 | 1.000 | -24.38 | 39.67 |  |  |
| **Squat (kg)** | Control | ISO | 12.913 | 9.110 | 0.508 | -10.53 | 36.36 |  |  |
|  |  | DEF | 10.674 | 9.332 | 0.792 | -13.34 | 34.69 |  |  |
|  | ISO | Control | -12.913 | 9.110 | 0.508 | -36.36 | 10.53 |  |  |
|  |  | DEF | -2.239 | 8.824 | 1.000 | -24.95 | 20.47 |  |  |
|  | DEF | Control | -10.674 | 9.332 | 0.792 | -34.69 | 13.34 |  |  |
|  |  | ISO | 2.239 | 8.824 | 1.000 | -20.47 | 24.95 |  |  |
| *Note:* Based on estimated marginal means. * The mean difference is significant at the ,05 level. b. Adjustment for multiple comparisons: Bonferroni. DEF, slight energy deficit; FFAT, fat-free adipose tissue; FM, fat mass; ISO, isocaloric; LST, lean soft tissue; LST–FFAT, LST adjusted for FFAT. | | | | | | | | | |

**Table S2.** Bonferroni-adjusted pairwise comparisons of total weekly training load

|  | | | **Mean Difference (I-J)** | **Std. Error** | ***p*^b^** | **95% Confidence Interval for Difference^b^** | |
| --- | --- | --- | --- | --- | --- | --- | --- |
|  |  |  |  |  |  | **Lower Bound** | **Upper Bound** |
| **LBTL** | **Control** | ISO | -63,375^*^ | 1.929 | <0.001 | -68.31 | -58.44 |
|  |  | DEF | -20,180^*^ | 1.877 | <0.001 | -24.98 | -15.38 |
|  | **ISO** | Control | 63,375^*^ | 1.929 | <0.001 | 58.44 | 68.31 |
|  |  | DEF | 43,194^*^ | 1.929 | <0.001 | 38.26 | 48.13 |
|  | **DEF** | Control | 20,180^*^ | 1.877 | <0.001 | 15.38 | 24.98 |
|  |  | ISO | -43,194^*^ | 1.929 | <0.001 | -48.13 | -38.26 |
| **UBTL** | **Control** | ISO | -28,400^*^ | 0.592 | <0.001 | -29.91 | -26.88 |
|  |  | DEF | -2,888^*^ | 0.576 | <0.001 | -4.36 | -1.41 |
|  | **ISO** | Control | 28,400^*^ | 0.592 | <0.001 | 26.88 | 29.91 |
|  |  | DEF | 25,511^*^ | 0.592 | <0.001 | 24.00 | 27.03 |
|  | **DEF** | Control | 2,888^*^ | 0.576 | <0.001 | 1.41 | 4.36 |
|  |  | ISO | -25,511^*^ | 0.592 | <0.001 | -27.03 | -24.00 |
| *Note:* Based on estimated marginal means. * The mean difference is significant at the ,05 level. b. Adjustment for multiple comparisons: Bonferroni. DEF, slight energy deficit; FFAT, fat-free adipose tissue; FM, fat mass; ISO, isocaloric; LST, lean soft tissue; LST–FFAT, LST adjusted for FFAT. | | | | | | | |
